# Loss of the interleukin-6 receptor causes immunodeficiency, atopy, and abnormal inflammatory responses

**DOI:** 10.1084/jem.20190344

**Published:** 2019-06-24

**Authors:** Sarah Spencer, Sevgi Köstel Bal, William Egner, Hana Lango Allen, Syed I. Raza, Chi A. Ma, Meltem Gürel, Yuan Zhang, Guangping Sun, Ruth A. Sabroe, Daniel Greene, William Rae, Tala Shahin, Katarzyna Kania, Rico Chandra Ardy, Marini Thian, Emily Staples, Annika Pecchia-Bekkum, William P.M. Worrall, Jonathan Stephens, Matthew Brown, Salih Tuna, Melanie York, Fiona Shackley, Diarmuid Kerrin, Ravishankar Sargur, Alison Condliffe, Hamid Nawaz Tipu, Hye Sun Kuehn, Sergio D. Rosenzweig, Ernest Turro, Simon Tavaré, Adrian J. Thrasher, Duncan Ian Jodrell, Kenneth G.C. Smith, Kaan Boztug, Joshua D. Milner, James E.D. Thaventhiran

**Affiliations:** 1 Medical Research Council Toxicology Unit, University of Cambridge, Cambridge, UK; 2 Department of Medicine, University of Cambridge, Cambridge Biomedical Campus, Cambridge, UK; 3 Ludwig Boltzmann Institute for Rare and Undiagnosed Diseases, Vienna, Austria; 4 CeMM Research Center for Molecular Medicine, Austrian Academy of Sciences, Vienna, Austria; 5 Sheffield Teaching Hospitals National Health Service Trust, Sheffield, UK; 6 Department of Infection Immunity and Cardiovascular Disease, University of Sheffield, Sheffield, UK; 7 Department of Haematology, University of Cambridge, Cambridge Biomedical Campus, Cambridge, UK; 8 National Institute for Health Research BioResource, Cambridge University Hospitals, Cambridge Biomedical Campus, Cambridge, UK; 9 Department of Biochemistry, Faculty of Biological Sciences, Quaid-i-Azam University, Islamabad, Pakistan; 10 Laboratory of Allergic Diseases, National Institute of Allergy and Infectious Diseases, National Institutes of Health, Bethesda, MD; 11 Cancer Research UK Cambridge Institute, Cambridge Biomedical Campus, Cambridge, UK; 12 Department of Dermatology, Sheffield Teaching Hospitals National Health Service Trust, Sheffield, UK; 13 Medical Research Council Biostatistics Unit, Cambridge Biomedical Campus, Cambridge, UK; 14 National Health Service Blood and Transplant Cambridge, Cambridge Biomedical Campus, Cambridge, UK; 15 Barnsley Hospitals National Health Service Foundation Trust, Barnsley, UK; 16 Immunology Department, Armed Forces Institute of Pathology, Rawalpindi, Pakistan; 17 Department of Laboratory Medicine, Clinical Center, National Institutes of Health, Bethesda, MD; 18 Herbert and Florence Irving Institute for Cancer Dynamics, Columbia University, New York, NY; 19 New York Genome Center, New York, NY; 20 Molecular and Cellular Immunology Section, University College London Great Ormond Street Institute of Child Health, Great Ormond Street Hospital National Health Service Trust, London, UK; 21 Department of Oncology, University of Cambridge, Cambridge Biomedical Campus, Cambridge, UK; 22 Department of Pediatrics and Adolescent Medicine, Medical University of Vienna, Vienna, Austria; 23 St. Anna Kinderspital and Children’s Cancer Research Institute, Department of Pediatrics and Adolescent Medicine, Medical University of Vienna, Vienna, Austria; 24 Vienna Center for Rare and Undiagnosed Diseases, Vienna, Austria

## Abstract

IL-6 excess is central to the pathogenesis of multiple inflammatory conditions and is targeted in clinical practice by immunotherapy that blocks the IL-6 receptor encoded by *IL6R*. We describe two patients with homozygous mutations in *IL6R* who presented with recurrent infections, abnormal acute-phase responses, elevated IgE, eczema, and eosinophilia. This study identifies a novel primary immunodeficiency, clarifying the contribution of IL-6 to the phenotype of patients with mutations in *IL6ST, STAT3*, and *ZNF341,* genes encoding different components of the IL-6 signaling pathway, and alerts us to the potential toxicity of drugs targeting the IL-6R.

## Abstract (precis)

Spencer et al. report the first description of human IL-6R deficiency in two patients presenting with recurrent infections, atopy, elevated IgE, and abnormal acute-phase responses.

The central role of IL-6 in the pathogenesis of inflammatory conditions such as rheumatoid arthritis, juvenile idiopathic arthritis, and giant cell arteritis is confirmed by the efficacy of tocilizumab, an anti–IL-6R antibody (Tanaka et al., 2018). IL-6 interacts with a multi-subunit receptor complex, initially by the binding of IL-6 to the 80-kD receptor (IL-6R), which exists in either membrane-bound or soluble forms. The IL-6/IL-6R complex ligates the signal-transducing subunit, glycoprotein 130 (GP130), leading to a wave of receptor, JAK, and STAT phosphorylation and culminating in the nuclear import of phosphorylated STAT (pSTAT) dimers, predominantly STAT3, that activate transcription, leading to growth and cellular differentiation (Taga and Kishimoto, 1997).

While the consequences of excessive IL-6 signaling in humans have been well established, the consequences of impairment have been more elusive. Reports of disease-causing neutralizing IL-6 antibodies have been informative (Puel et al., 2008); however, those reports would benefit from the corroboration and specificity that the study of genetic loss provides. Loss-of-function (LOF) mutations affecting GP130 and STAT3 lead to multisystem disorders encompassing IgE elevation, infection susceptibility, and connective tissue abnormalities (Grimbacher et al., 1999; Schwerd et al., 2017); however, the specific contribution of IL-6 remains undefined. Studies of patients with loss of IL-6R function therefore provide a unique opportunity to establish the role for IL-6 in these disorders and define how impaired IL-6 signaling impacts human health.

## Results and discussion

### Clinical findings

**Figure 1.**
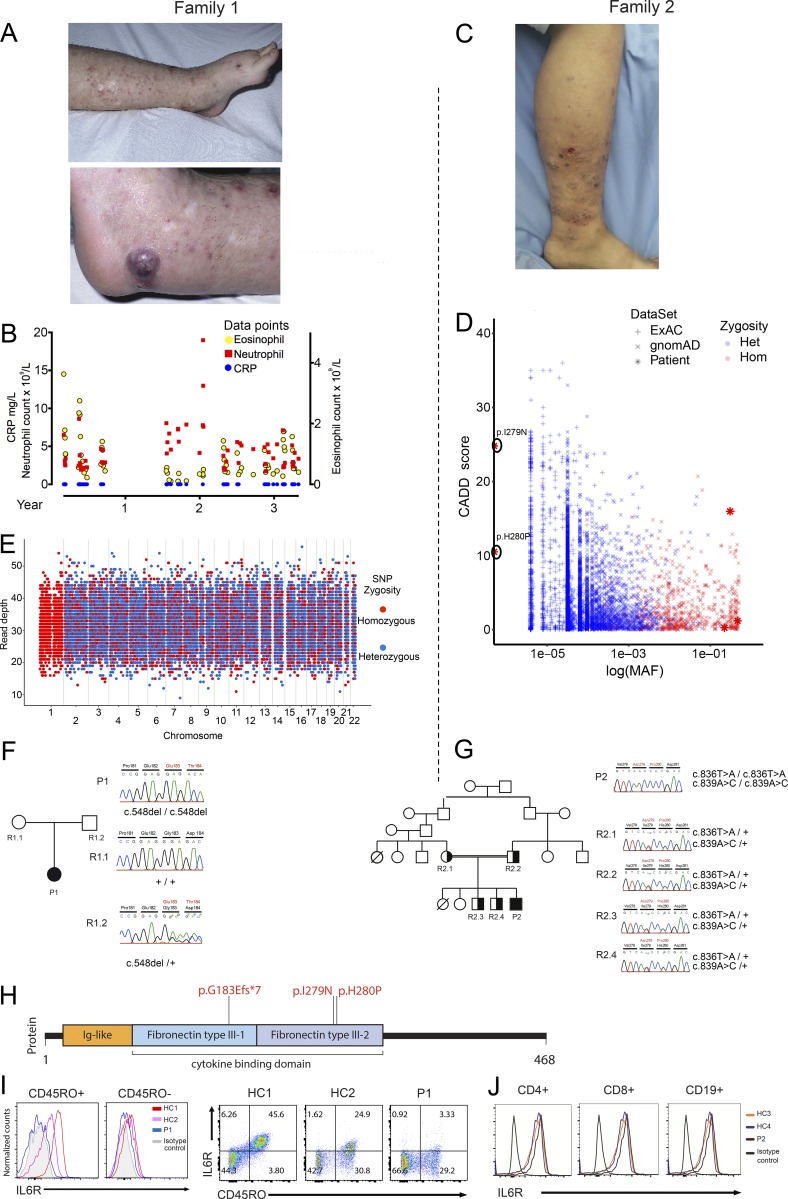
**Skin lesions, pedigrees, genetic sequencing results, and IL-6R expression levels in two patients with immunodeficiency, atopy, and abnormal inflammatory responses. (A)** Photographs of the skin lesions of P1 demonstrating the excoriation and skin-colored nontender subcutaneous nodules (top) and a typical abscess (bottom) with the notable lack of surrounding erythema. **(B)** P1 diagnostic laboratory measurements of CRP and neutrophil and eosinophil count measured during multiple infective episodes over a 3-yr period. Normal ranges: CRP <5 mg/liter, neutrophils 1.7–6.5 × 10^9^/liter, eosinophils 0.04–0.5 × 10^9^/liter. **(C)** Photograph of the excoriating eczematous dermatitis found on the lower extremities of P2. **(D)** CADD/MAF graph of the variants identified in the *IL6R* gene of P2, plotted with the variants in the populations extracted from the databases ExAC and gnomAD. **(E)** Plot of read depth and zygosity at 23,368 genome-wide autosomal SNV positions in P1’s WGS data. Red dots, homozygous SNV calls; blue dots, heterozygous SNV calls. **(F and G)** The pedigrees, dideoxy sequencing chromatograms, and IL-6R mutations of P1 (F) and P2 (G). **(H)** Schematic representation of the IL-6R protein together with the positions of the mutations identified in P1 and P2. **(I)** Left: Fluorescence histograms of IL-6R expression within the memory (CD45RO^+^) and naive (CD45RO^−^) populations of gated CD3^+^CD4^+^ T cells from P1. Right: Dot-plots of IL-6R and CD27 of gated CD3^+^CD4^+^ T cells from P1. **(J)** Fluorescence histograms of IL6R expression on CD4^+^ and CD8^+^ T cells and B cells (CD19^+^) of P2. HC, healthy control.

We identified two patients with atopic dermatitis, elevated IgE, reduced inflammatory responses, and recurrent skin and lung infections. Patient 1 (P1; 29-yr-old female), who was born to white English parents of European ancestry without known consanguinity, initially presented with neonatal mastitis. She suffered from recurrent sinopulmonary infections including *Haemophilus influenza* pneumonia and recurrent deep staphylococcal skin infections. She also suffered from asthma and moderate atopic dermatitis, affecting lower legs, thighs, buttocks, and elbows. The dermatitis was associated with extensive subcutaneous nodules that were intensely pruritic, and persistent scratching led to development of deep excoriations on legs, buttocks, arms, shoulders, and scalp. Apart from one episode of impetiginized varicella infection in her first year, no significant viral infections were noted. Total IgE level was elevated, with the highest recorded level being 7,060 kU/liter (normal 0–81 kU/liter). She had mildly reduced IgG, IgA, and IgM. A striking feature was a lack of clinically adequate inflammatory response in the face of marked infection. While she did develop minor nontender lymphadenopathy in a number of infections, her abscesses were notable for their lack of erythema (Fig. 1 A) and pyrexia. A recordable fever was observed on only two occasions, throughout multiple inpatient admissions for systemic infection. In addition, the peripheral neutrophil response was typically minimal in proportion to her illnesses, and infectious episodes were more marked by eosinophilia than neutrophilia (Fig. 1 B). Further, despite gross elevations in serum IL-6 levels (Table 1), which stimulates the serological acute phase response (Majello et al., 1990; Hutchinson et al., 1994), C-reactive protein (CRP) was never detectable during infection or convalescence on standard diagnostic assay (detection level 0.175 mg/liter). Patient 2 (P2; 15-yr-old male) was born to consanguineous parents of Pakistani origin. From 6 mo of age, he suffered from relapsing-remitting, itchy, eczematous skin lesions (with no other history of clinical allergy, severity scoring of atopic dermatitis: 61.85) which were managed with topical fusidic acid and betamethasone (Fig. 1 C). The family history was remarkable for the early deaths of a sibling and a cousin, who were both affected by similar skin lesions, but no further details are known. In his first 2 yr of life, P2 had recurrent upper respiratory tract infections and two pneumonias, requiring hospitalization and i.v. antibiotic treatment. At age 10, he developed a methicillin-resistant *Staphylococcus aureus* psoas abscess requiring surgical drainage. He also had recurrent skin abscesses; however, his CRP was barely above the limits of detection (highest recording 2.1 mg/liter), even during acute inflammatory episodes. Serum biochemical parameters and liver and kidney function tests were within normal limits. No viral or fungal infections were noted. Neutrophilia was observed in blood counts during abscess episodes, and IgG, IgA, and IgM levels were mildly reduced; in contrast, IgE was elevated (787 kU/liter, normal range 0–100 kU/liter). At present, he receives regular i.v. immunoglobulin replacement and antibiotic prophylaxis, alleviating his symptoms.

**Table 1.** Immunological characteristics of patients

	**P1**		**P2**
	**Age 6 yr**	**Age 26 yr**	**Age 14 yr**
Absolute lymphocyte count (×10^9^/liter)	2.4 (1.1–4.5)	1.74 (1.1–4.5)	1.48 (1.1–4.5)
CD3^+^ T cells			
Absolute (×10^9^/liter)	1.7 (0.75–2.51)	1.43 (0.75–2.51)	1.11 (0.75–2.51)
%	71 (55–84)	82.3 (55–84)	75 (55–84)
CD4^+^ T cells			
Absolute (×10^9^/liter)	0.94 (0.43–1.69)	0.82 (0.43–1.69)	0.66 (0.43–1.69)
%	39 (31–60)	47.1 (31–60)	45 (31–60)
CD8^+^ T cells			
Absolute (×10^9^/liter)	0.67 (0.22–1.21)	0.52 (0.22–1.21)	0.47 (0.22–1.21)
%	28 (13–41)	30.1 (13–41)	32 (13–41)
CD16^+^/CD56^+^ NK cells			
Absolute (×10^9^/liter)	0.12 (0.12–0.60)	0.09 (0.12–0.60)	0.07 (0.12–0.60)
%	5 (5–27)	4.9 (5–27)	5 (5–27)
CD19^+^ B cells			
Absolute (×10^9^/liter)	0.48 (0.12–0.64)	0.23 (0.12–0.64)	0.21 (0.12–0.64)
%	20 (6–25)	13.3 (6–25)	14 (6–25)
IgE (KU/liter)	**859** (0–48)	**7,060** (0–81)	**795** (0–81)
IgG (g/liter)	**4.5** (4.9–16.1)	7.71 (6–16)	**5.3** (6–16)
IgG1 (g/liter)	4 (2.3–6.4)		
IgG2 (g/liter)	0.28 (0.4–4.5)		
IgG3 (g/liter)	0.78 (0.1–1.1)		
IgG4 (g/liter)	<0.02 (0–0.8)		
IgM (g/liter)	0.5 (0.5–1.9)	**0.24** (0.5–1.9)	**0.3** (0.5–1.9)
IgA (g/liter)	**0.3** (0.4–2.0)	0.91 (0.8–2.8)	**0.6** (0.8–2.8)
Hib IgG (µg/ml)	**<0.15** (prevaccine)^a^	3.01^a^	
Pneumococcal titer (IU/ml)		**38**^b^	
Tetanus IgG (IU/ml)	**>0.1**^c^		
NBT (%)	0/96		100
DHR			
Resting (%)		6 (Control, 2)	
Stimulated (%)		100 (Control, 100)	
IL-6 (pg/ml)		**830.8** (0–7)	
IL-6 (pg/ml)		**205.6** (0–7)	

Abnormal results are shown in bold. Numbers in parentheses indicate reference values of the corresponding measurements. DHR, dihydrorhodamine; Hib, *H. influenza* bacteria; NBT, nitroblue tetrazolium.

a Minimum protective level, 0.15 µg/ml; optimum protective level, 1.0 µg/ml.

b A value of 60 represents the 25th centile of the normal population.

c Minimum protective level, 0.01 IU/ml; optimum protective level, 0.1 IU/ml; long-term protective level, >1.0 IU/ml.

A more detailed patient history for P1 is provided in Materials and methods. The results of clinical laboratory testing, including immunoglobulin levels and lymphocyte subsets from both patients, are provided in Table 1.

### Genetic and functional assessment

Assessment of common single-nucleotide polymorphisms in the whole-genome sequencing (WGS) data of P1 demonstrated a rare genetic anomaly: chromosome 1 (C1) contained only homozygous variants (Fig. 1 E). Since read depth was even across all chromosomes (excluding large genomic deletions), the C1 loss of heterozygosity is explained by uniparental isodisomy, when both homologous chromosomes are inherited from the same parent. C1 contained a set of rare coding homozygous variants (Table S2) with one priority candidate: a 1-bp frameshift deletion (NM_000565.3:c.548delG; p.G183Efs*7) in exon 4 of the *IL6R* gene (Fig. 1 F) that leads to a premature stop codon. For P2, whole-exome sequencing identified two homozygous missense variants in exon 6 of *IL6R* (ENST00000368485.3:c.836T>A, p.I279N, combined annotation-dependent depletion [CADD] score 24.8, and ENST00000368485.3:c.839A>C, p.H280P, CADD score 10.5). Both variants were ultrarare, had high CADD scores (Fig. 1 D), relative to previously observed homozygous *IL6R* variants, and are predicted to be damaging because their CADD scores are above the mutation significance cutoff (Itan et al., 2016) of 3.313 (the lower 99% CI of CADD-based mutation significance cutoff for *IL6R* variants, estimated using pathogenic mutations reported in Human Gene Mutation Database and ClinVar). The H280 amino acid is at (and the I279 adjacent to) the face of IL-6R’s D2 convex bulge that docks into the D3 domain of GP130. Since this interface is necessary for the IL-6/IL-6R complex’s GP130 affinity (Boulanger et al., 2003), disruption due to amino acid substitution at these sites could limit IL-6 signaling. Both variants were confirmed by Sanger sequencing and appropriately segregated under the assumption of autosomal recessive inheritance (Fig. 1, F and G). The candidate variants in P1 and P2 (Fig. 1 H) are novel and not found in the aggregate 150,000 human genomes available from the Genome Aggregation Database (gnomAD), Trans-Omics for Precision Medicine, and National Institute for Health Research (NIHR) BioResource–Rare Diseases (NBR-RD).

**Figure 2.**
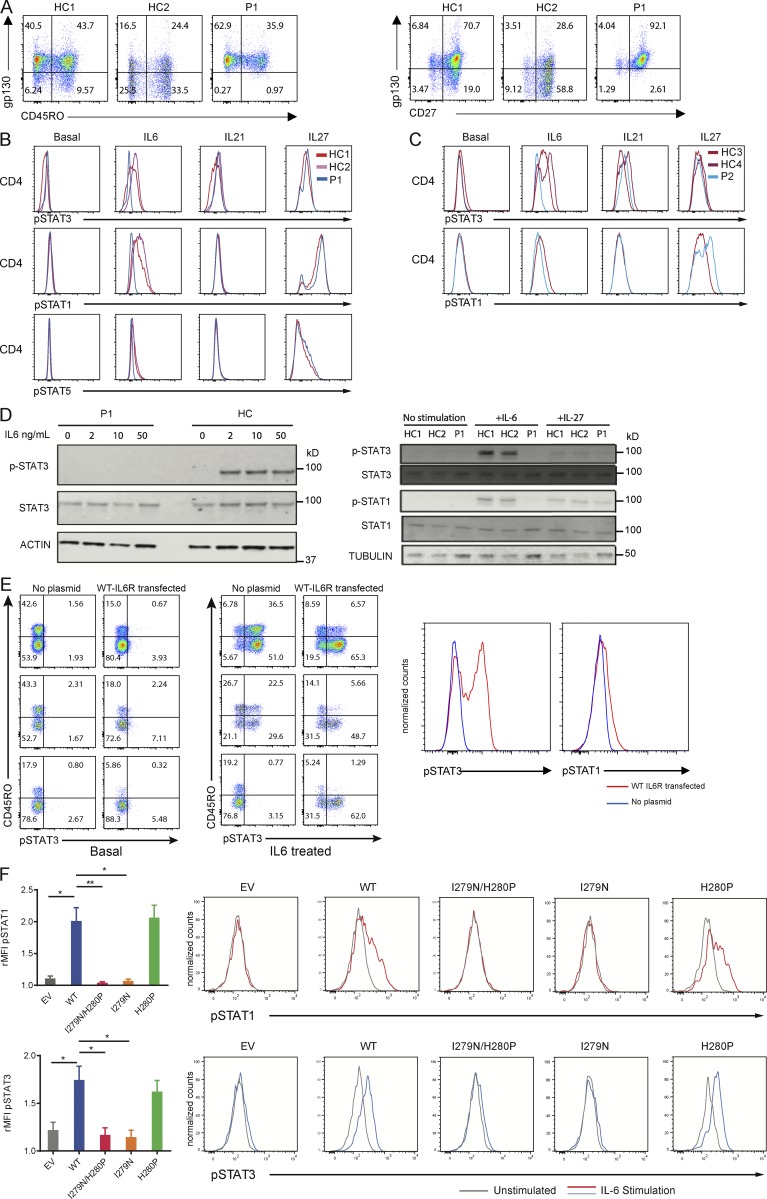
**STAT signaling responses with and without ectopic expression of IL-6R in two patients with *IL6R* mutations. (A)** Left: Dot plots of GP130 and CD45RO of gated CD3^+^CD4^+^ T cells from P1 and two healthy controls (HCs). Right: Dot plots of gp130 and CD27 of gated CD3^+^CD4^+^ T cells from P1. **(B)** Fluorescence histograms of the indicated anti-pSTAT proteins’ expression in CD4^+^ T cells from healthy controls and P1 upon stimulation. **(C)** Fluorescence histograms of the indicated anti-pSTAT proteins’ expression in CD4^+^ T cells from healthy controls and P2. **(D)** Left: Western blot of pSTAT3, STAT3, and actin as a loading control in P1 and healthy control PBMCs stimulated with the indicated IL-6 concentration for 30 min. Right: Western blot of pSTAT3, STAT3, pSTAT1, STAT1, and tubulin as a loading control in patient and healthy controls. PBMCs stimulated with the indicated cytokine concentrations for 30 min. **(E)** Left: pSTAT3 in healthy controls’ and IL-6R–null (P1) CD4^+^ T cells, transfected with a vector expressing WT IL-6R at baseline and after IL-6 stimulation. Right: The overlaid fluorescence histograms of pSTAT3 and pSTAT1 in PBMCs from the IL-6R–null patient in WT-IL-6R transfected and untransfected cells stimulated with IL-6. **(F)** Bar graphs indicating relative mean fluorescence intensity (rMFI) of pSTAT1 and pSTAT3 upon IL-6 stimulation of HEK293 T cells transfected with WT IL-6R, p.I279N/p.H280P IL6-R, p.I279N IL6-R, p.H280P IL6-R, or an empty vector (EV). Representative histograms are shown for each condition. Data represent two replicates of three independent experiments (unpaired two-tailed Student’s *t* test done on mean of technical replicates; *, P < 0.05; **, P < 0.01). Error bars represent SEM.

We next tested the effect of the mutations on the expression and function of IL-6R. The homozygous premature stop of P1 led to reduced surface IL-6R expression on CD45RO^+^ memory CD4^+^ T cells and CD27^−^ memory CD4^+^ T cells, in comparison to healthy control expression (Fig. 1 I). By contrast, surface IL-6R expression was present on peripheral blood lymphocytes of P2 (Fig. 1 J). Despite P2’s normal receptor expression, IL-6–mediated phosphorylation of STAT3 and STAT1 proteins was absent in P1 and barely detectable in P2, contrasting with a normal response to IL-27 and IL-21 stimulation (Fig. 2, B–D). GP130 expression on peripheral blood lymphocytes of P1 was actually increased compared with control (Fig. 2 A), likely due to the absence of IL-6–mediated down-regulation (Wang et al., 1998). This result therefore additionally demonstrates that a level of tonic IL-6 signaling occurs in healthy controls. Given that IL-6 and IL-27 both signal through GP130 (Zhong et al., 1994; Pflanz et al., 2004), the STAT-signaling data confirmed normal GP130 signal transduction and localized the lesion to IL-6R in both patients.

To demonstrate that the lack of IL-6R in P1 causes the absent IL-6–pSTAT response, an expression construct with WT IL-6R was transfected into the peripheral blood mononuclear cells (PBMCs) of P1 and healthy controls, demonstrating equal surface IL6R expression (Fig. S1) and restoring IL-6–dependent phosphorylation of STAT3 and STAT1 (Fig. 2 E). To investigate the pathogenicity of the two homozygous variants identified in P2, because of the limited primary tissue available, HEK293 cells were used. As two homozygous variants had been identified, to identify which was the causative mutation, cells were transfected with WT, p.I279N, p.H280P, or p.I279N/p.H280P vectors (the latter including both variants in the same plasmid). IL-6–induced STAT1 and STAT3 phosphorylation was deficient in p.I279N-transfected cells, whereas the responses were normal in WT and p.H280P transfectants, demonstrating that the p.I279N variant, rather than p.H280P variant, was pathogenic (Fig. 2 F). Of note, while an I279D substitution completely abrogated IL-6 binding to IL-6R (Yawata et al., 1993), staining for IL-6R in transfected cells showed that although Flag-tagged protein levels were equivalent in I279N and WT, IL-6R staining was substantially reduced (Fig. S1). The discrepancy in surface staining between the ex vivo and transfected cells could be explained by the presence of soluble IL-6R bound to patient PBMCs, which could contribute to the normalized staining ex vivo, whereas the transfection experiments express only membrane-bound IL-6R. There could also be other posttranscriptional processing differences in the overexpression system which may be altered uniquely by the I279N mutation.

### Immunophenotyping

**Figure 3.**
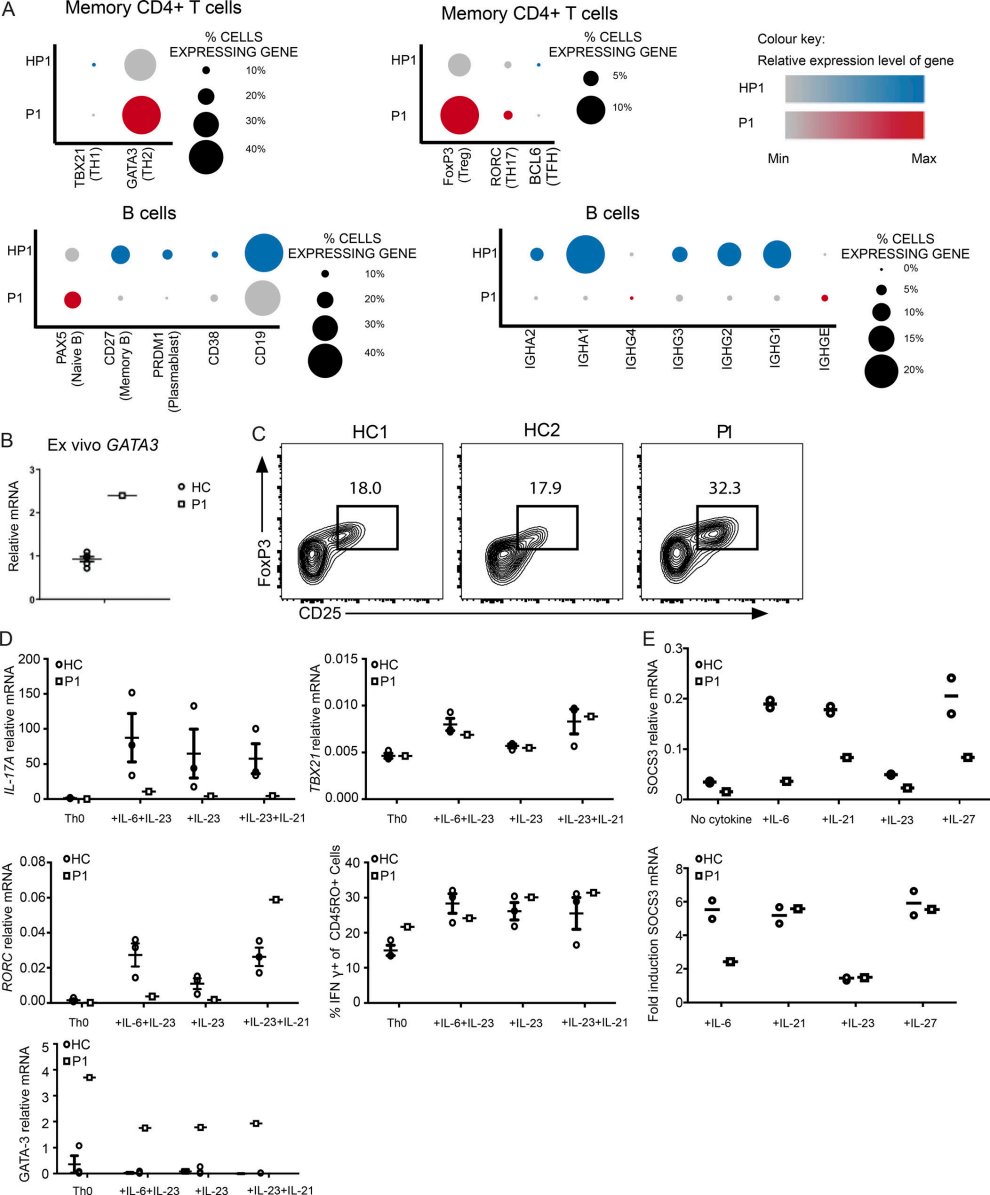
**Immunophenotype of IL-6R–null patient. (A)** Split dot plot visualizations of how gene expression varies in P1 and her healthy father (HP1) generated from analysis of single-cell RNA-seq data from PBMCs. Top: Lineage defining transcription factor expression in memory CD4^+^ T cells. Bottom: Expression of genes indicating state of B lymphocyte differentiation and immunoglobulin isotype class in B cells. The size of the dot corresponds to the percentage of cells expressing the gene, and the color represents the average expression level. **(B)** GATA3 mRNA levels in ex vivo PBMCs isolated from P1 and healthy controls (HCs), detected by real-time PCR. **(C)** Flow cytometry analysis of CD25^+^FOXP3^+^ cells of P1 and two healthy controls, gated on the CD3^+^CD4^+^CD45RO^+^CD127^−^ compartment. **(D)** Naive cells from healthy controls (*n* = 3) and P1 were cultured under the indicated Th17-inducing conditions for 6 d. *IL-17A*, *RORC*, *GATA3*, and *TBX21* mRNA levels were detected by real-time PCR. **(E)** *SOCS3* mRNA levels of naive cells from P1 and healthy controls activated by the indicated cytokines for 18 h. Error bars represent SEM.

The above results defining P1 as IL-6R null and P2 with protein-positive IL-6R-LOF provided a unique opportunity to investigate the contribution of IL-6 to the immunological phenotype. Single-cell RNA sequencing (RNA-seq) of mononuclear cells isolated from P1 compared with a normal familial control showed an increased proportion of memory CD4^+^ T cells expressing T helper 2 (Th2)–associated *GATA3*, as well as higher expression levels of GATA3 (Fig. S2) (Fig. 3 A). Similar results were observed via real-time PCR measurement of *GATA3* expression in P1 PBMCs (Fig. 3 B), as well as activated naive CD4 T cells (Fig. 3 D). P1 had reduced numbers of class-switched memory B cells, and within this population a higher proportion of IgE- and IgG4-expressing B cells was found (Fig. 3 A). P1 also had an increased proportion of FOXP3^+^ regulatory T cells, with increased relative expression of *FOXP3* (Fig. 3 A), and this was confirmed on flow cytometry (Fig. 3 C). Interestingly, P1 naive CD4^+^ T cell precursors poorly up-regulated *IL17A* compared with controls (Fig. 3 A), under all conditions. Induction remained impaired in cultures with IL-23 and IL-21, despite the observation that IL-23 and IL-21 led to a normal increase in the expression of *RORC*, *T-bet*, and IFNγ (Fig. 3 D). The impairment was present even in the condition of IL-23 addition without IL-6, potentially because IL-23R must be up-regulated by a STAT3 agonist (de Wit et al., 2011). The impairment was not due to a global transcriptional unresponsiveness to these cytokines since, in addition to the *RORC* up-regulation, the fold increase with stimulation was comparable to controls, despite the lower absolute basal and induced levels of STAT3-target, *SOCS3*, induced by IL-21, IL-23, and IL-27 (Fig. 3 E). These results demonstrate a nonredundant role for IL-6 in the in vitro induction of the expression of *IL17A*, but not *RORC* from naive precursors. It is likely that in vivo, given the presence of Th17 cells and absence of fungal infections dependent on IL-17, there are redundant cytokines that allow for Th17 differentiation in the absence of IL-6 signaling.

**Figure 4.**
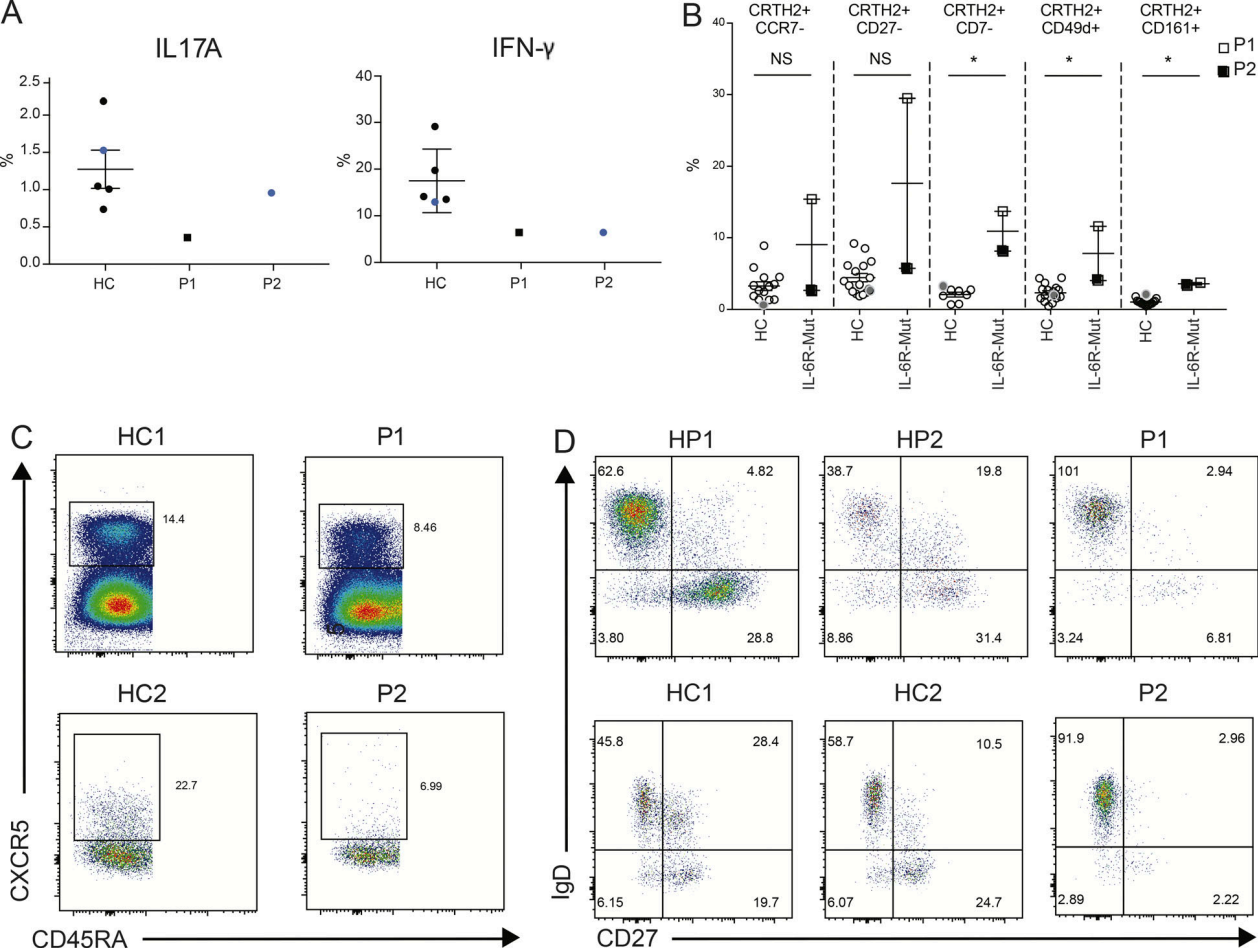
**Immunophenotype of patients with *IL6R* mutations. (A)** Percentages of IL-17A–producing (left) and IFNγ-producing (right) CD4^+^ T cells from healthy controls (HCs; *n* = 5), P1 (dark square), and P2 (blue circle) from two independent experiments. Black, data from experiment 1; blue, data from experiment 2. **(B)** Flow cytometry analysis of expression of markers found in pathological effector Th2 cells in CRTH2^+^ memory CD4^+^ T cells. Mann–Whitney *U* test; *, P < 0.05. **(C)** Flow cytometry analysis of blood CXCR5^+^ memory cTFHs in P1, P2, and healthy controls. **(D)** Flow cytometry assessment of B lymphocyte class switching and memory cell formation in P1, P2, and healthy controls and the healthy parents (HP). Error bars represent SEM.

We proceeded to confirm which aspects of P1’s phenotype were shared with P2. While both patients and controls had comparable production of IL-17A, they both had reduced production of IFNγ (Fig. 4 A). The elevated GATA3 seen in the single-cell, PBMC, and naive T-cell induction experiments from P1 was not associated with increased CCR4^+^ (correlated to Th2 cells) levels, or any striking increase in the production of Th2 cytokines IL-4 or IL-13 ex vivo (Fig. S3). However, pathological effector Th2 cells, which are more closely associated with Th2-associated tissue pathology (Mitson-Salazar et al., 2016; Wambre et al., 2017), were elevated in both patients, although more markedly in P1, consistent with the higher IgE (Fig. 4 B). While an expansion of such proatopic T cells is consistent with the clinical phenotype, this result appears initially surprising because of data implicating IL-6 in generating Th2 cells (Rincón et al., 1997) and promoting IgE class switching (Vercelli et al., 1989; Jabara et al., 1990). However, STAT3 activation leads to the expression of and physical binding to ERBIN, encoded by the gene *ERBB2IP*, which limits TGF-β signaling by cytoplasmic sequestering of the SMAD2/3 components and normally prevents expression of IL-4Rα on naive CD4^+^ T cells and B cells, restraining Th2 responses (Lyons et al., 2017). Corroborating this was the observation that P1’s *IL4R* expression was elevated in these cell types in the single-cell RNA-seq analysis (Fig. S3). So, IL-6 may have opposing roles in Th2 responses, in that it can promote IL-4 transcription (Diehl et al., 2002) and IgE class switching but could prevent IL-4 receptor expression by blocking TGF-β signaling.

In animal models, IL-6 is essential for circulating T follicular helper (cTFH) cell induction (Eto et al., 2011) and the observation of decreased cTFH cells in patients with STAT3-LOF (Ma et al., 2016) prompted this assessment in P1 and P2. Both patients had a reduced percentage of cTFH cells compared with healthy controls (Fig. 4 C). Furthermore, IL-6 (initially termed as the B cell differentiation factor) promotes B cell differentiation, and both patients had reduced CD27^positive^ memory B cells and reduced class-switched memory B cells (Fig. 4 D).

Aspects of these patients’ clinical phenotype are shared by patients with mutations in *STAT3*, *IL6ST* (Schwerd et al., 2017; Shahin et al., 2019), or *ZNF341* (Béziat et al., 2018; Frey-Jakobs et al., 2018), which are part of a series of disorders associated with IgE elevation (Table S1). ZNF341 deficiency leads to impaired STAT3 levels and signaling, primarily in the hematopoietic compartment, and is associated with atopy, variable elevation of IgE, and susceptibility to *Candida* and staphylococcal infections. The similarities to the *IL6ST-mutant* patient are greater, as the GP130-deficient patients presented with recurrent staphylococcal and streptococcal infections and a marked reduction of an acute-phase response, but with eosinophilia and elevation of their IgE. In addition, similar to IL-6R deficiency described here, fungal infections were not a feature, in keeping with diminished but present circulating Th17 cells seen in these patients. However, like *STAT3* LOF patients, the affected *IL6ST* patients also demonstrated skeletal and connective tissue defects, such as craniosynostosis and scoliosis, possibly caused by GP130-dependent cytokines such as IL-11 (Schwerd et al., 2017). The IL-6 link with the atopic phenotype of this group of patients had been suggested by the association between common polymorphisms in the *IL6R* that diminish its function and increase both levels of IgE (Wang et al., 2016) and susceptibility to asthma (Ferreira et al., 2013). Furthermore, B cell–specific deletion of STAT3 is sufficient to elevate serum IgE in mice (Kane et al., 2016). The results from the IL-6R-LOF patients therefore clarify the contribution of deficient IL-6 signaling to the phenotype of patients with *ZNF341*, *GP130*, and *STAT3*-LOF mutations. This includes elevated IgE, atopic dermatitis, and a susceptibility to staphylococcal infections. This may indicate novel therapeutics for the alleviation of these symptoms: for example, exogenous recombinant soluble IL-6R could increase the presentation of IL-6 to GP130.

Although results from murine studies have suggested that IL-6 signaling is an obligate necessity for a normal acute-phase response, we have lacked confirmatory evidence for this in humans. STAT3-LOF patients and GP130 patients have reduced but detectable acute-phase proteins, including CRP (Freeman and Holland, 2009; Schwerd et al., 2017), while patients with anti–IL-6 autoantibodies, who present with skin infections similar to the patients presented here, may also have other neutralizing autoantibodies preventing CRP induction (Puel et al., 2008). This report demonstrates that human CRP production is directly dependent on IL-6 signaling and therefore that a recordable CRP in a tocilizumab-treated patient indicates incomplete IL-6 blockade. In addition to basic insights into inborn errors of the IL-6/GP130/STAT3 axis, the patients described in this report provide clear evidence for the scope of potential side effects of IL-6 blockade. Furthermore, they highlight the consequences of inborn loss of IL-6 signaling, corroborating the intriguing observation that very early introduction of tocilizumab (<2 yr of age), unlike in adults, is associated with substantially increased immediate-type hypersensitivity reactions (Mallalieu et al., 2017).

## Materials and methods

### Detailed case history

P1 was born postterm after a normal pregnancy, to nonconsanguineous white English parents of European ancestry. Her birthweight was 2.8 kg (9th–25th centile). She was admitted for 2 wk with apyrexial neonatal mastitis within the first month, and within the first year of life she had suffered from multiple infections, including impetiginized varicella and right and left lower lobe pneumonias, and was noted to have painless lymphadenopathy. She received standard childhood vaccinations, including diphtheria/tetanus/pertussis, polio, and measles/mumps/rubella, without complications. She continued to grow normally on height and weight centile charts, always above the 50th centile.

Having been managed initially by her local district general hospital, she was referred to a specialist immunology service at the age of 5 yr with *H. influenza* pneumonia associated with right lower lobe collapse, and a right ear infection, which responded to antibiotics. Nontender cervical and inguinal lymphadenopathy was noted; biopsy of 6–10-mm inguinal lymph nodes demonstrated reactive changes but was notable for an excess of eosinophils, and cultures grew *S. aureus*. She responded to treatment with i.v. flucloxacillin, and after this she was commenced antibiotic prophylaxis with cotrimoxazole 480 mg once a day; however, purulent rhinitis and upper respiratory tract symptoms persisted despite prophylaxis. She received *H. influenza B* and pneumococcal vaccination (Pneumovax) at age 6 without complication. Antibodies to measles, mumps, rubella, varicella, tetanus, and diphtheria were found to be present. On the basis of her elevated IgE levels, a putative diagnosis of hyper-IgE syndrome was given, with an National Institutes of Health hyper-IgE syndrome score retrospectively calculated at 45. However, IgE levels subsequently fluctuated and on a few occasions were normal.

Over the following 12 yr, the patient continued to present with recurrent sinopulmonary infections. A wheeze often accompanied chest symptoms, and she was started on steroid inhalers after a diagnosis of asthma was established. Recurrent otitis externa and otitis media eventually became chronic and were associated with scarring of the tympanic membrane. She also had two admissions with cellulitis requiring i.v. antibiotics. It was noted that with episodes of infection, although she felt systemically unwell, with nausea and vomiting, a recordable fever was observed on only two occasions, throughout multiple inpatient admissions for systemic infection. In addition, neutrophil response was blunted, and she did not generate a CRP increase. Response to antibiotics tended to be slow or absent. However, throughout this time she was found to be thriving and missed very little school.

At the age of 11, she developed a severe pneumonia that was complicated by a pleural effusion. Cultures from blood and pleural fluid grew fully sensitive *S. aureus*; however, the pleural fluid aspirated was notably lacking pus. Around this time, she also began to develop occasional skin spots and skin-colored nontender subcutaneous nodules, which were associated with dermatitis and intense pruritus. Persistent scratching led to the development of deeply excoriated craters on thighs, buttocks, scalp, arms, and shoulders. Histology obtained from biopsy of an eczematous lesion on the buttock excluded vasculitis, granulomatous disease, or pemphigoid but revealed an underlying eosinophil-rich inflammatory dermatosis, severe enough to extend into the subcutaneous fat, with flame figure formation. However, a definitive histopathological diagnosis could not be established. Skin and nasal swabs routinely grew *S. aureus*, which was usually fully sensitive, although methicillin-resistant *S. aureus* was grown on several occasions. She has had multiple admissions with cellulitis requiring i.v. antibiotics since childhood. She also had occasional episodes of moderate, transient oral and vaginal candida, which occurred in association with antibiotic treatment; however, she has never had disseminated or invasive *Candida* infection.

Repeated attempts at prophylaxis with numerous antibiotics were unsuccessful, although patient adherence may have been unsatisfactory. Staphylococcal eradication therapy also failed. She transitioned to adult care, but thereafter she was lost to follow up between the ages of 18 and 26 yr.

She represented to immunology at the age of 26 yr, at which point all her immunological tests were repeated. Notable abnormal results were significantly elevated IgE, eosinophilia, absent mannose-binding lectin, and high levels of baseline IL-6 (830.8 pg/ml and 205.6 pg/ml on two samples; normal range, 0–7 pg/ml).

Further respiratory tract infections since representation have been associated with growth of *H. influenzae*, *Moraxella catarrhalis*, and *Escherichia coli*. She has had one episode of peripherally inserted central catheter line–associated sepsis, with *S. aureus* grown from blood cultures and *Proteus* and *S. aureus* cultured from the line tip. Skin lesions persisted and included deep excoriations, subcutaneous nodules, and eczema, with pruritus as a predominant symptom. In adulthood, she has had recurrent admissions several times a year with painful swelling of her left leg, shoulder, arm, and hand. Subcutaneous and perifascial edema without collections are usually seen in the limbs. Aspiration of the shoulder collection has been attempted, but never successfully. More recently, she presented with recurrent mastitis and a lump in the left breast, which was felt to be an abscess with associated ductal disease; on the penultimate episode, 8 ml of pus growing *S. aureus* was aspirated from an abscess inferior to the left lateral malleolus, showing that pus formation is not completely absent. The most recent episode was of severe cellulitis of the entire left leg, with pain, erythema, marked swelling, and edema involving the subcutis down to and including the fascial plane on magnetic resonance imaging, showing that erythema is not completely absent; however, the patient remained apyrexial with undetectable CRP throughout.

### Clinical studies

The NBR-RD study was established in the UK to further the clinical management of patients with rare diseases by providing a national resource of WGS data. Written informed consent was obtained from the patients and their relatives or healthy normal donors, in accordance with the principles of the ethics committees of the Institutional Review Boards of the Medical University of Vienna (EK-Nr:499/2011) and the East of England–Cambridge South National Institutional Review Board (13/EE/0325).

### Genetic analysis

For P1, WGS was performed by Illumina. The sample was fragmented to 450-bp fragments, processed with the Illumina TruSeq DNA PCR-Free Sample Preparation kit, and sequenced using 150-bp reads on a single HiSeqX lane. The delivered genome had a minimum 15× coverage over ≥95% of the reference autosomes. Illumina performed the alignment to GRCh37 genome build and calling of variants <50 bp in length using Isaac software, while large deletions were called with Manta and Canvas algorithms. The WGS data files were received at the University of Cambridge High Performance Computing Service for further in-house quality control and processing. The analyses presented here are based on single nucleotide variants (SNVs) and indels (<50 bp long) with overall pass rate (OPR) >0.98 across 13,037 in-house NBR-RD genomes, and high-confidence deletion calls. Variants were annotated with Sequence Ontology terms according to their predicted consequences; their frequencies in other genomic databases (gnomAD, UK10K, 1000 Genomes), if they have been associated with a disease according to the Human Gene Mutation Database Pro database; and internal metrics (allele number, allele count, allele frequency [AF], and OPR).

For assessment of ancestry, relatedness, and loss of heterozygosity, a set of reliably genotyped, unlinked SNVs were chosen as follows: 292,878 autosomal SNVs typed by three widely used Illumina genotyping arrays were filtered to exclude those with at least one missing genotype in the NBR-RD 13,037 genomes or OPR <0.99; more than two alleles in the 1000 Genomes Phase 3 or NBR-RD datasets; and minor AF (MAF) <0.3 in NBR-RD. Finally, after pruning with PLINK so that all pairs of SNVs had r^2^ <0.2, we were left with 32,875 SNVs to be used for further analyses. Ancestry relative to 1000 Genomes populations was estimated by performing principal component analysis. PC-Relate R package and the same set of SNVs were used to generate overall proportion of genome-wide homozygosity, as well as pairwise kinship coefficients that allowed us to compute a maximal unrelated sample set for the purposes of internal AF estimation. A denser set of SNVs (all with OPR >0.98 and internal AF >0.5%) and bcftools roh tool were used to compute the exact regions displaying loss of heterozygosity, which were found to span the entire C1 and account for the total homozygosity in this sample. The plot in Fig. 1 D is based on a subset of 23,368 SNVs that had nonreference genotype calls in P1 and was generated using ggbio R package (Yin et al., 2012). Nonsynonymous and splice-site SNVs and indels on C1 were filtered based on gnomAD AFs <0.001 and CADD score >20. Resulting variants are listed in Table S2, and the one in the *IL6R* was prioritized based on clinical presentation. We did not identify any rare large deletions or rare coding homozygous compound heterozygous SNVs and indels outside of C1.

For P2, whole-exome sequencing was performed using the TrueSeq Rapid Exome kit, Illumina HiSeq3000 system, and the cBot cluster generator. Burrows-Wheeler Aligner was used to align reads against version 19 of the human genome as a reference. Insertion-deletion realignment was performed as well as recalibration based on Genome Analysis Toolkit quality scores. To call SNVs and deletion-insertion variants, UnifiedGenotyper and Genome Analysis Toolkit Variant quality score recalibration was performed as described previously with minor modifications. Generated lists of SNVs and deletion-insertion variants were annotated with SNPEFF. Variants present in 1000 Genomes Project and/or gnomAD datasets with MAF ≥0.01 were excluded from further analyses. An internal database of >2,000 exomes was also used to filter out recurrent variants. In the resulting list of variants, under the assumption of autosomal recessive inheritance, homozygous changes were then subjected to prioritization using the SIFT, PolyPhen-2, and CADD tools to assess in silico the effect of the identified mutations (Table S2). All of the variants identified in *IL6R* gene of P2 were plotted in the CADD/MAF graph, including the population variants extracted from ExAC and gnomAD databases (Fig. 1 D).

Next-generation sequencing data are deposited at the European Genome-phenome Archive, which is hosted by the European Bioinformatics Institute and the Centre for Genomic Regulation, under accession IDs EGAD00001005051 (P1) and EGAS00001003675 (P2). The data are accessible through the relevant Data Access Committee via formal application at the European Genome-phenome Archive.

For P1, Sanger confirmation was performed using primers (forward, 5′-TGT​CCC​GTC​TTG​AGT​CTG​TG-3′; reverse, 5′-TGC​TTT​ATC​ATT​GCC​ACA​CC-3′) that amplified a 364-bp fragment spanning exon 4 of the *IL-6R* gene. For P2, forward, 5′-GGG​GCA​GGG​ACT​TTC​TGC-3′, and reverse, 5′-GTA​CTG​CGG​TGG​GCA​CTG​A-3′, primers were used to amplify exon 6.

### Single-cell RNA-seq

Single-cell RNA-seq libraries were prepared in the Cancer Research UK Cambridge Institute Genomics Core Facility using the following: Chromium Single Cell 5′ Library & Gel Bead Kit, PN-1000006; Chromium Single Cell A Chip Kit, PN-1000009; Chromium i7 Multiplex Kit, PN-120262; Chromium Single Cell V(D)J Enrichment Kit, Human B Cell, PN-1000016; Chromium Single Cell V(D)J Enrichment Kit, Human T Cell, PN-1000005; and the Single-Cell V(D)J Reagent Kits User Guide (Manual Part CG000086 Rev J; 10X Genomics).

Cell suspensions of both samples were resuspended in PBS-0.04% BSA and loaded into Chromium microfluidic chips with 10X Genomics 5′ V(D)J and gene expression chemistry to generate single-cell gel-bead emulsions using the Chromium controller (10X Genomics) according to the manufacturer’s recommendations. Suspensions containing ∼16,000 cells were loaded on the instrument with the expectation of collecting up to 9,000 gel-bead emulsions containing single cells (expected multiplet rate ∼7%). RNA from the barcoded cells for each sample was subsequently reverse-transcribed in a T100 Thermal cycler (Bio-Rad), and all subsequent steps to generate single-cell libraries were performed according to the manufacturer’s protocol with no modifications, with 14 cycles used for cDNA amplification. Then ∼50 ng of cDNA was used for gene expression library amplification by 14 cycles in parallel with cDNA enrichment and library construction for T and B cell libraries.

Library quality was confirmed with Agilent TapeStation 4200 (High Sensitivity D1000 ScreenTape to evaluate library sizes) and BMG LABTECH Clariostar Monochromator Microplate Reader (Invitrogen Quant-iT dsDNA Assay Kit; high sensitivity to evaluate dsDNA quantity). Each sample was normalized to equal molar concentration and pooled in the following ratio: 90% gene expression library, 5% human B cell library, 5% human T cell library. Samples were sequenced on an Illumina HiSeq 4000 as 2 × 150 paired-end reads, one full lane per pool (before analysis, gene expression data were trimmed to 26 bp, read 1; 8 bp, i7 index; and 98 bp, read 2).

Using the 10X Genomics Single Cell Immune Profiling solution, we obtained single cell V(D)J measurements and 5′ gene expressions for both P1 and her healthy father. To find the shared correlation structure of the combined father and P1 normalized and scaled gene expression matrices, we used canonical correlation analysis (Butler et al., 2018). The two datasets were then aligned using a dynamic time-warping algorithm as implemented in the Seurat R package (https://CRAN.R-project.org/package=Seurat). We then performed clustering on all the cells using a shared nearest neighbor modularity optimization–based clustering algorithm (Xu and Su, 2015).

By merging the gene expression data with the V(D)J counts, we were able to annotate TCR- and B cell receptor–bearing cells and to identify cell types, which clusters were memory CD4^+^ T cells, and which were B cell clusters. We confirmed this by examining cell type marker genes that were conserved between samples in each cluster.

RNA-seq data for P1 and her healthy father have been deposited in the ArrayExpress database at European Molecular Biology Laboratory/European Bioinformatics Institute under accession no. E-MTAB-8034.

### PAGE and Western blot analysis

Protein lysates were separated by SDS-PAGE and transferred onto a nitrocellulose membranes. Individual proteins were detected with antibodies against STAT3 (clone 124H6), STAT1 (clone 9H2), pSTAT3 (clone D3A7), pSTAT1 (clone D4A7), and β3-Tubulin (D65A4; Cell Signaling Technology). Secondary antibodies were either goat anti-mouse-IgG IRDye 680CW or goat anti-rabbit-IgG IRDye 800CW (LI-COR Biosciences). Quantification of bound antibodies was performed on an Odyssey Infrared Imaging system (LI-COR Biosciences).

### Flow cytometry analysis

For intracellular cytokine detection, cells were stimulated with 20 ng/ml PMA and 1 µM ionomycin for 5 h. 10 µg/ml brefeldin A was added after 2.5 h. Cells were then fixed and permeabilized with Cytofix/Cytoperm (BD Biosciences) per the manufacturer’s protocol and stained with Live/Dead Fixable Aqua dye, CD3 AF700, CD4 BUV395, IL-17F AF488, IFNγ fluorescein v450, IL-4 PE-Cy7 (BD Biosciences), CD45RO ECD (Beckman Coulter), and IL-17A PE and IL-21 e660 (eBiosciences). For P2, we conducted the same procedure with the following antibodies: CD3 APCH7, CD8 V500, CD45RA AF700, CD25 PE, IL4 APC, CD197 PE-CF594 (BD Biosciences), CD4 PCy7 (Beckman Coulter), IFNγ FITC, Il17A eFluor450 (eBiosciences), and CCR6 BV605 (Biozym Scientific). Intracellular pSTAT analysis was previously described in Shahin et al. (2019). Regulatory T cell staining was based on the eBioscience FOXP3 intracellular staining reagents and protocol. Other intracellular staining was done by fixation in PFA followed by methanol permeabilization. Additional antibodies used for flow cytometry analysis: IL-6R PerCP-Cy5.5 (BioLegend), CRTH2 AF647 (BD Biosciences), CCR6 BV421 (BD Biosciences), CXCR3 PE-Cy7 (BioLegend), CCR4 BV605 (BioLegend), CD27 BV650 (BioLegend), CCR7 BV711 (BioLegend), CD49d BV510 (BioLegend), CD161 FITC (BioLegend), CD7 PerCP-Cy5.5 (BioLegend), CD45RO ECD (Beckman Coulter), and Live/Dead Fixable Blue (Life Technologies). Additional antibodies used for P2: IL6R PE (BD Biosciences), CD7 PerCPCy5.5 (BD Biosciences), CD161 FITC (BD Biosciences), CD45RO AF700 (BD Biosciences), CRTH2 AF647 (BD Biosciences), CXCR3 BV711 (BioLegend), CCR6 BV605 (BioLegend), CD4 V500 (BD Biosciences), CD27 V450 (BD Biosciences), CCR4 PeCy7 (BD Biosciences), CCR7 PE-CF594 (BD Biosciences), and CD49d PE (BD Biosciences).

### Real-time PCR

To assay *SOCS3* up-regulation in response to cytokine stimulation, naive CD4 T cells were stimulated with or without IL-6 (100 ng/ml; Peprotech), IL-21 (100 ng/ml; Peprotech), IL-23 (100 ng/ml; R&D Systems), or IL-27 (50 ng/ml; R&D Systems) for 18 h. Cells were then washed and pelleted. Total RNA was extracted from PBMCs with the RNeasy Mini Kit (Qiagen). For real-time PCR, 1 µg of total RNA was reverse transcribed (Invitrogen), and the resulting cDNA was amplified by means of PCR with the ABI 7500 Sequencer and TaqMan expression assays (Applied Biosystems). 18S was used as a normalization control. The data were analyzed with the 2^−ΔΔCT^ method, and results are expressed as mean fold induction.

### Th17 differentiation

Naive CD4^+^ T cells were isolated from PBMCs using negative selection magnetic-activated cell sorting microbeads (Miltenyi Biotec) per the manufacturer’s protocol. Purity was >95% in all samples run. For T cell differentiation, cells were stimulated at 100,000 cells/well in 96-well flat-bottom plates that had been coated with anti-CD3 (10 µg/ml; OKT3 eBioscience) antibodies. The cells were cultured in X-VIVO 15 medium at 37°C/5% CO_2_ for 6 d in the presence of soluble anti-CD28 (0.5 ug/ml; BD PharMingen). For Th17, anti-IFNγ (10 µg/ml; BD PharMingen) and IL-1β (10 ng/ml; Peprotech) were added to all the groups, and IL-23 (100 ng/ml; R&D Systems), IL-6, and IL-21 (both 20 ng/ml; Peprotech) were also added as indicated. For Th0, 100 U/ml IL-2 (R&D Systems) was added.

### Transfection of PBMCs

PBMCs from P1 and healthy volunteers were nucleofected with pcDNA3.1^+^/C-(K)-DYK plasmid DNA expressing WT IL-6R using a human T cell nucleofector kit (VPA-1002; Lonza) according to the manufacturer’s protocol for unstimulated human T cells (program V-024). Cells were cultured in T cell medium (RPMI 1640 supplemented with 2 mM l-glutamine and 10% FBS) overnight before assays, transfected T cells expressing WT IL-6R were identified as Flag^+^ cells by anti-Flag antibody staining.

### Transfection of HEK293T cells

HEK293T cells were plated on 96-well flat-bottom plates and transfected with pcDNA3.1^+^/C-(K)-DYK plasmid DNA expressing WT IL-6R, p.I279N/p.H380P IL-6R, p.I279N IL-6R, p.H280P IL-6R, or an empty plasmid using the Effectene reagent (Qiagen) according to the manufacturer’s protocol. Cells were cultured in DMEM (10% FBS and 2mM l-glutamine) and incubated overnight. Transfected cells were then starved for 3 h in serum-free DMEM followed by the pSTAT assay described above.

### Online supplemental material

Fig. S1 shows an assessment of IL-6R expression in cells ectopically expressing IL-6R or IL-6R mutants. Fig. S2 shows t-distributed stochastic neighbor embedding (tSNE) projections of the IL-6R–null patient and her father’s PBMCs. Fig. S3 shows ex vivo FACS assessment of T cell differentiation in P1 and healthy controls. Table S1 compares clinical features of patients with *IL6R* mutations and other patient cohorts with defined gene defects presenting with elevated IgE. Table S2 lists homozygous nonsynonymous variants in P1 and P2 identified by WGS.

## Supplementary Material

Supplemental Materials (PDF)
