# Supplementary material for: Loss of the interleukin-6 receptor causes immunodeficiency, atopy, and abnormal inflammatory responses
Source: J Exp Med. 2019 Jun 24;216(9):1986–98. doi: 10.1084/jem.20190344 (PMC6719421; doi:10.1084/jem.20190344)
Supplement: Supplemental Materials (PDF) [file JEM_20190344_sm.pdf]

JEM

Supplemental material

Spencer et al., https://doi.org/10.1084/jem.20190344

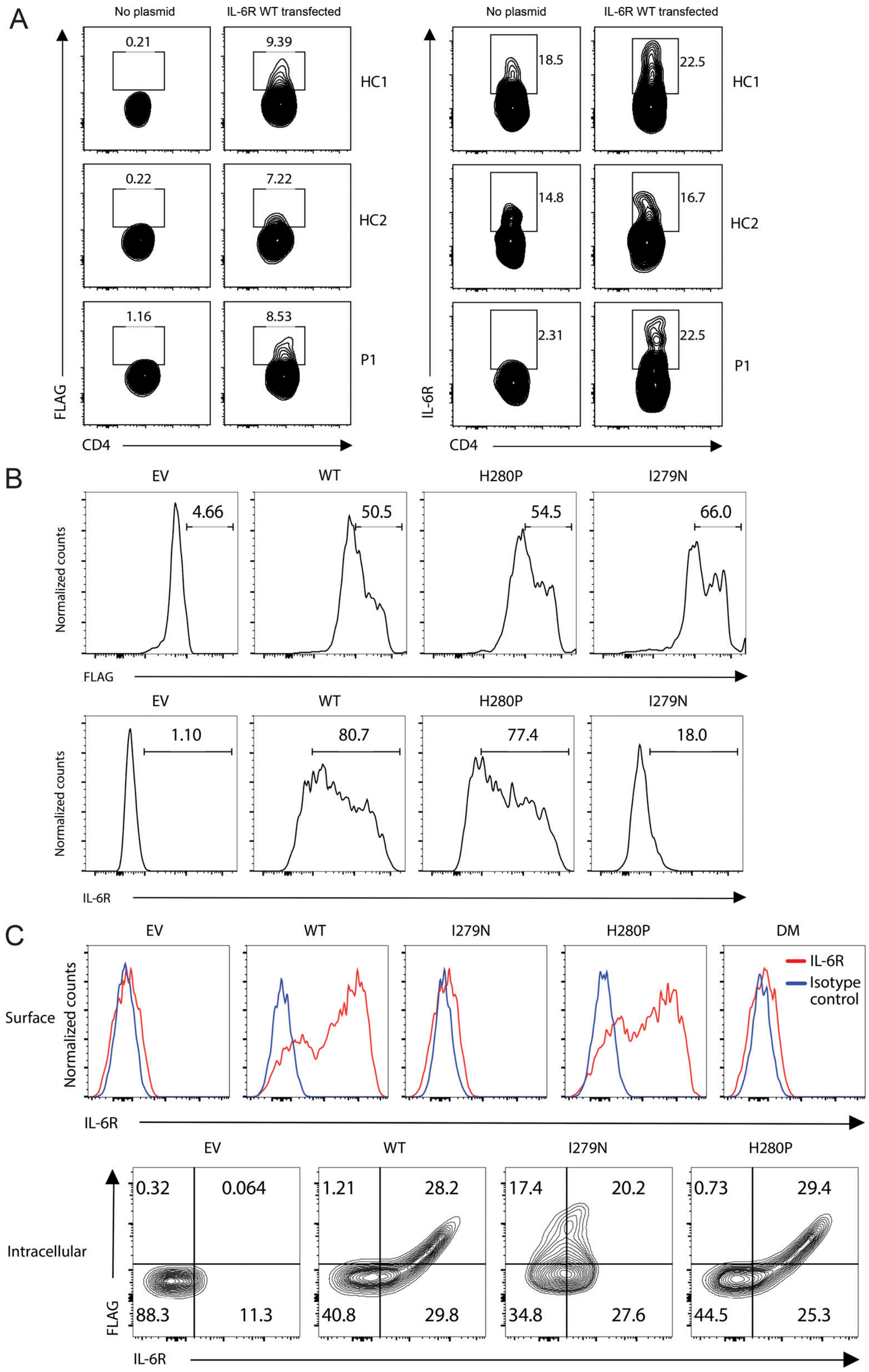

Figure S1. **Assessment of ectopic IL6R expression. (A)** PBMCs from healthy controls (HC) and P1 were isolated from Ficoll gradient and transfected with WT IL-6R cDNA cloned in the pcDNA3.1^{+}/C-(K)-DYK plasmid using T cell nucleofection. After 24 h, the PBMCs were intracellularly stained with anti-CD4, anti-FLAG, and anti–IL-6R antibodies and analyzed by flow cytometry. **(B)** HEK293 cells were transfected with empty vector (EV), WT, H280P, and I279N mutants cloned in the pcDNA3.1^{+}/C-(K)-DYK plasmid. After 24 h, the proteins and transfection efficiencies were measured by flow cytometry using anti-FLAG and IL-6R antibody (UV4). Similar results were observed when stained with the IL-6R clone M5 (not depicted). **(C)** Human healthy PBMCs were transfected with empty vector (EV), WT, I279N, H280P, and I279N/H280P (DM) mutants using T cell nucleofection. After 24 h, the proteins and transfection efficiencies were measured by flow cytometry using anti-FLAG and IL-6R antibody (UV4). Top: Surface staining (red, IL-6R; blue, isotype control). Bottom: Intracellular staining.

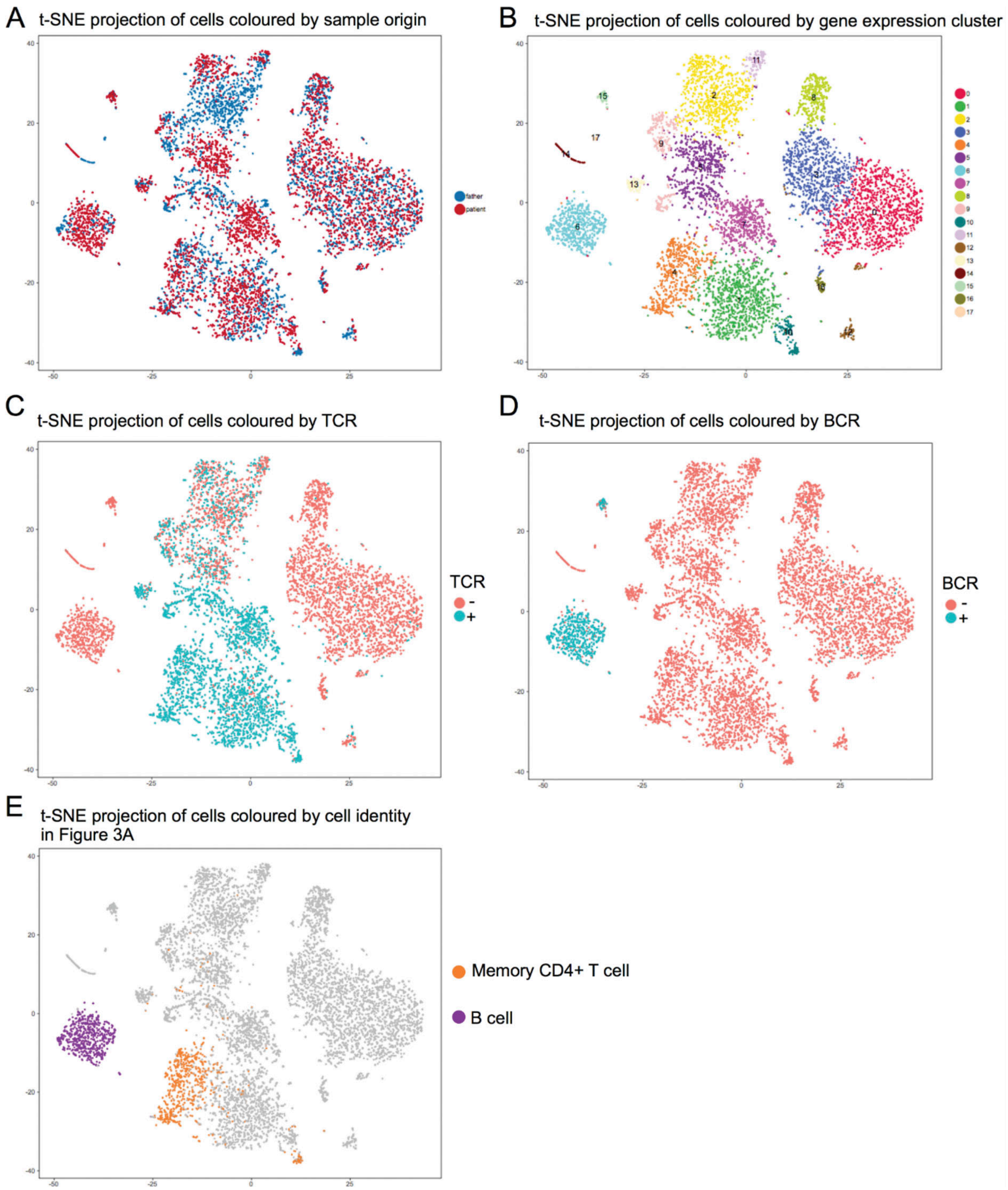

Figure S2. **tSNE projections of PBMCs from the IL-6R–null patient (P1) and her father (HP1). (A–D)** Clusters of single cells are identified using a shared-nearest-neighbor graph (Xu and Su, 2015). Each cell is assigned a cluster. The clustering groups together cells that have similar expression profiles. tSNE plots are color coded by sample (A), gene expression cluster membership (B), TCR presence (C), and B cell receptor (BCR; D). **(E)** Memory CD4⁺ T and B cells were identified by gene expression or TCR or BCR presence and assigned for the differentiation analysis displayed in Fig. 3 A.

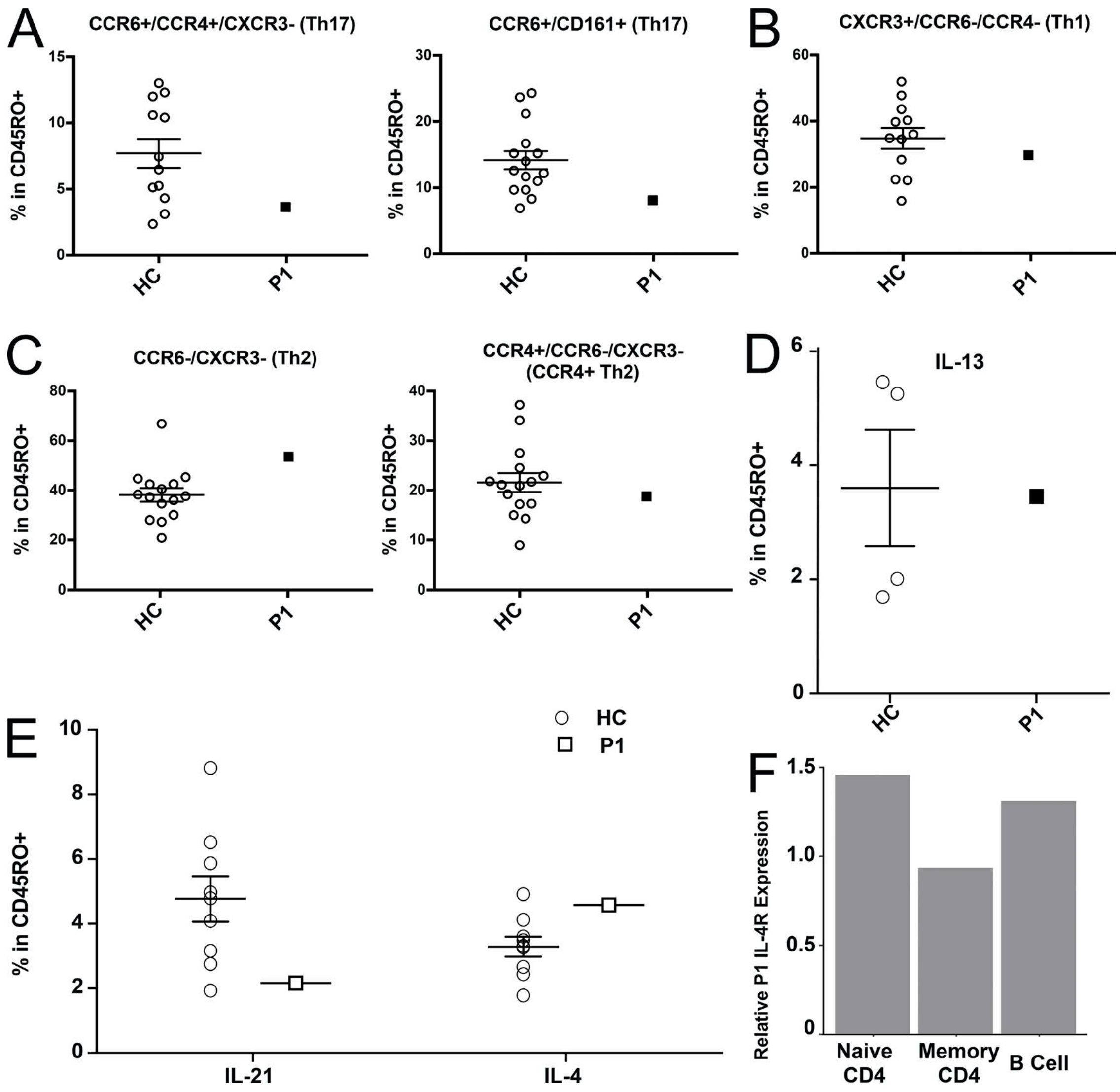

Figure S3. **Phenotype assessment of P1. (A–C)** FACS assessment of the indicated surface markers used to identify memory CD4+ Th17 (A), Th1 (B), and Th2 (C) T cells. **(D and E)** FACS analysis of the intracellular cytokine staining of healthy control (HC) and P1 PBMCs after PMA and ionomycin stimulation for IL-21 and IL-4 (D) and IL-13 (E). **(F)** Relative average expression of *IL4R* in the patient with respect to the father, within naive CD4+ and memory CD4+ T cell and B cell populations. For each cluster, mean *IL4R* expression was calculated for patient and father cells. These values were divided to obtain the fold-change of average expression between the two samples. Error bars represent SEM.

Table S1. **Comparative analysis of clinical features of patients with *IL6R* mutations and other patient cohorts with defined gene defects presenting with elevated IgE**

	IL6R	***IL6ST***	***STAT3***	***ZNF341***	***DOCK8***	***CARD11***	***PGM3***	***ERBIN***
n	2	2	140	19	64	12	29	3
Inheritance	AR	AR	AR	AR	AR	AD	AR	AD
IgE	↑[a]	↑[a]	↑↑[a]	↑↑[a]	↑[a]	↑[a]	↑↑[a]	↑[a]
Eosinophilia	+	+	+	+/−	+	+	+	+
Eczema	++	+	+	+	++	++	+	+
Infections								
Abscess	++	−/+	++	+	+	+	++	−
Pneumonia	++	++	+++	+	+	++	++	++
Sinusitis/otitis	+	+	+	+	++	+	+	+
Candidiasis	−	−	++	++	+	+	+	−
Viral infections	−	−	+	−	+++	+	++	−
Musculoskeletal abnormalities	−	++	++	+	+	+/−	−	−
IgE hypersensitivity	−	+	+	+	+++	+++	+++	+
Autoimmunity	−	−	+	−	++	+	++	−
CNS abnormalities	−	++	+	+	++	−	++	−
Neoplasia	−	−	+	+/−	++	−	+	−
Immunological features								
Immunoglobulins	Normal/low	Normal	Normal	Normal/high IgG	Normal/low	Normal/low	Normal	Normal
Lymphopenia	−	−	−	+/−	+/−	−	+	−
Th17 cells	Normal/low	Normal/low	Low	Low	Low	Normal	Normal	Normal
CD19$^+$ B cells	Normal	Normal/low	Normal/low	Normal	Normal/low	Normal/low	Normal/low	Normal
Switched-memory B cells	Low	Normal/low	Low	Low	Low	Normal/low	Normal/low	Normal

Recently a comprehensive table comparing phenotypes with elevated IgE was also presented in Shahin et al., 2019. AD, autosomal dominant; AR, autosomal recessive; CNS, central nervous system.

[a]↑↑ refers to IgE levels >5,000 IU/ml; ↑ refers to IgE levels 1,000–5,000 IU/ml.

Table S2. **Filtered homozygous C1 variants identified in P1 and filtered homozygous variants in P2**

Gene	Gene description	Coding effect	Genomic annotation (GRCh37)	cDNA annotation	Protein annotation	gnomAD AF	CADD score
P1							
MAP7D1	MAP7 domain containing 1	Missense	chr1:g.36641812A>G	ENST00000316156.4: c.863A>G	ENSP00000320228.4: p.Gln288Arg	0.00003237	17.7
PIK3R3	Phosphoinositide-3-kinase regulatory subunit 3	Stop gain	chr1:g.46642005G>A	ENST00000540385.1: c.139C>T	ENSP00000439913.1: p.Arg47*	0.00001619	31.0
CCDC18	Coiled-coil domain containing 18	Stop gain	chr1:g.93711681G>T	ENST00000343253.7: c.2998G>T	ENSP00000343377.7: p.Glu1000*	0.00001247	50.0
IL6R	Interleukin-6 receptor	Frameshift	chr1:g.154407084del	ENST00000368485.3: c.548del	ENSP00000357470.3: p.Gly183Glufs*7	0	NA
NES	Nestin	Missense	chr1:g.156639266C>T	ENST00000368223.3: c.4714G>A	ENSP00000357206.3: p.Glu1572Lys	0	21.1
PLXNA2	Plexin A2	Missense	chr1:g.208218435G>A	ENST00000367033.3: c.3616C>T	ENSP00000356000.3: p.Leu1206Phe	0.00004329	23.6
KCTD3	Potassium channel tetramerization domain containing 3	Missense	chr1:g.215792524C>T	ENST00000259154.4: c.1777C>T	ENSP00000259154.2: p.Leu593Phe	0.00000813	24.6
P2							
MAB21L3	Mab21-like-3	Splice donor	chr1:g.116666979G>T	ENST00000369500.3: c.481+1G>T	ENST00000369500.3: c.481+1G>T	0.000125	33.0
CYP26A1	Cytochrome p450	Missense	chr10:g.94836739G>A	ENST00000224356.4: c.1172G>A	ENST00000224356.4: c.1172G>A	0.000398	31.0
TECTA	Tectorin alpha	Missense	chr11:g.121016276C>T	ENST00000392793.1: c.3556C>T	ENST00000392793.1: c.3556C>T	0.000874	28.3
SLC25A24	Solute carrier family 25	Splice donor	chr1:g.108742577C>A	ENST00000565488.1: c.183+1G>T	ENST00000565488.1: c.183+1G>T	0	28.1
PEAR1	Platelet endothelial aggregation receptor	Missense	chr1:g.156877958A>G	ENST00000338302.3: c.941A>G	ENST00000338302.3: c.941A>G	0.0000258	27.5
TRIM69	Tripartite motif containing protein	Missense	chr15:g.45050880T>C	ENST00000559390.1: c.641T>C	ENST00000559390.1: c.641T>C	0.00000406	27.2
ARHGEF12	Rho guanine nucleotide exchange factor 12	Missense	chr11:g.120328462G>A	ENST00000397843.2: c.2222G>A	ENST00000397843.2: c.2222G>A	0.000252	27.0
IL6R	Interleukin 6 receptor alpha	Missense	chr1:g.154408473T>A	ENST00000368485.3: c.836T>A	ENST00000368485.3: c.836T>A	0	24.8
DIXDC1	DIX-domain containing 1	Missense	chr11:g.111845662A>G	ENST00000440460.2: c.611A>G	ENST00000440460.2: c.611A>G	0.000399	24.3
SPTA1	Spectrin alpha	Missense	chr1:g.158585147A>G	ENST00000368147.4: c.6647T>C	ENST00000368147.4: c.6647T>C	0.0000122	23.8
DLG5	Discs large 5	Missense	chr10:g.79577595C>T	ENST00000372391.2: c.3724G>A	ENST00000372391.2: c.3724G>A	0.0000196	23.8
STARD9	Start domain containing protein 9	Missense	chr15:g.43009197C>T	ENST00000290607.7: c.13445C>T	ENST00000290607.7: c.13445C>T	0.000859	23.2
SORBS1	SORBIN and SH3 domains containing protein 1	Missense	chr10:g.97096822G>C	ENST00000371247.2: c.3095C>G	ENST00000371247.2: c.3095C>G	0.000834	23.1
OR6K3	Olfactory receptor family 6 subfamily K member 3	Missense	chr1:g.158687688T>A	ENST00000368145.1: c.218A>T	ENST00000368145.1: c.218A>T	0	22.5
ALG8	Alpha-1,3-glucosyltransferase	Missense	chr11:g.77812075C>T	ENST00000299626.5: c.1516G>A	ENST00000299626.5: c.1516G>A	0.000427	20.9
WDR47	WD repeat domain 47	Missense	chr1:g.109538319T>C	ENST00000400794.3: c.1598A>G	ENST00000400794.3: c.1598A>G	0.0000487	20.4
SV2A	Synaptic vesicle glycoprotein 2A	Missense	chr1:g.149878301T>G	ENST00000369146.3: c.1786A>C	ENST00000369146.3: c.1786A>C	0.0000651	19.0

Table S2. **Filtered homozygous C1 variants identified in P1 and filtered homozygous variants in P2 (*Continued*)**

Gene	Gene description	Coding effect	Genomic annotation (GRCh37)	cDNA annotation	Protein annotation	gnomAD AF	CADD score
ASTN2	Astrotactin 2	Missense	chr9:g.120177057C>T	ENST00000361209.2: c.160G>A	ENST00000361209.2: c.160G>A	0	18.8
IFT81	Intraflagellar transport 81	Missense	chr12:g.110566928A>G	ENST00000242591.5: c.422A>G	ENST00000242591.5: c.422A>G	0.000359	18.3
SOX4	SRY-Box4	In-frame deletion	chr6: g.21595443_21595445del	ENST00000244745.1: c.678_680del	ENST00000244745.1: c.678_680del	0.00006620	16.7
EMILIN1	Elastin microfibril interfacer 1	Missense	chr2:g.27306843G>A	ENST00000380320.4: c.2404G>A	ENST00000380320.4: c.2404G>A	0	15.9
IL6R	Interleukin 6 receptor alpha	Missense	chr1:g.154408476A>C	ENST00000368485.3: c.839A>C	ENST00000368485.3: c.839A>C	0	10.5

References

Shahin, T., D. Aschenbrenner, D. Cagdas, S.K. Bal, C.D. Conde, W. Garncarz, D. Medgyesi, T. Schwerd, B. Karaatmaca, P.G. Cetinkaya, et al. 2019. Selective loss of function variants in *IL6ST* cause Hyper-IgE syndrome with distinct impairments of T-cell phenotype and function. *Haematologica.* 104:609–621. https://doi.org/10.3324/haematol.2018.194233

Xu, C., and Z. Su. 2015. Identification of cell types from single-cell transcriptomes using a novel clustering method. *Bioinformatics.* 31:1974–1980. https://doi.org/10.1093/bioinformatics/btv088
